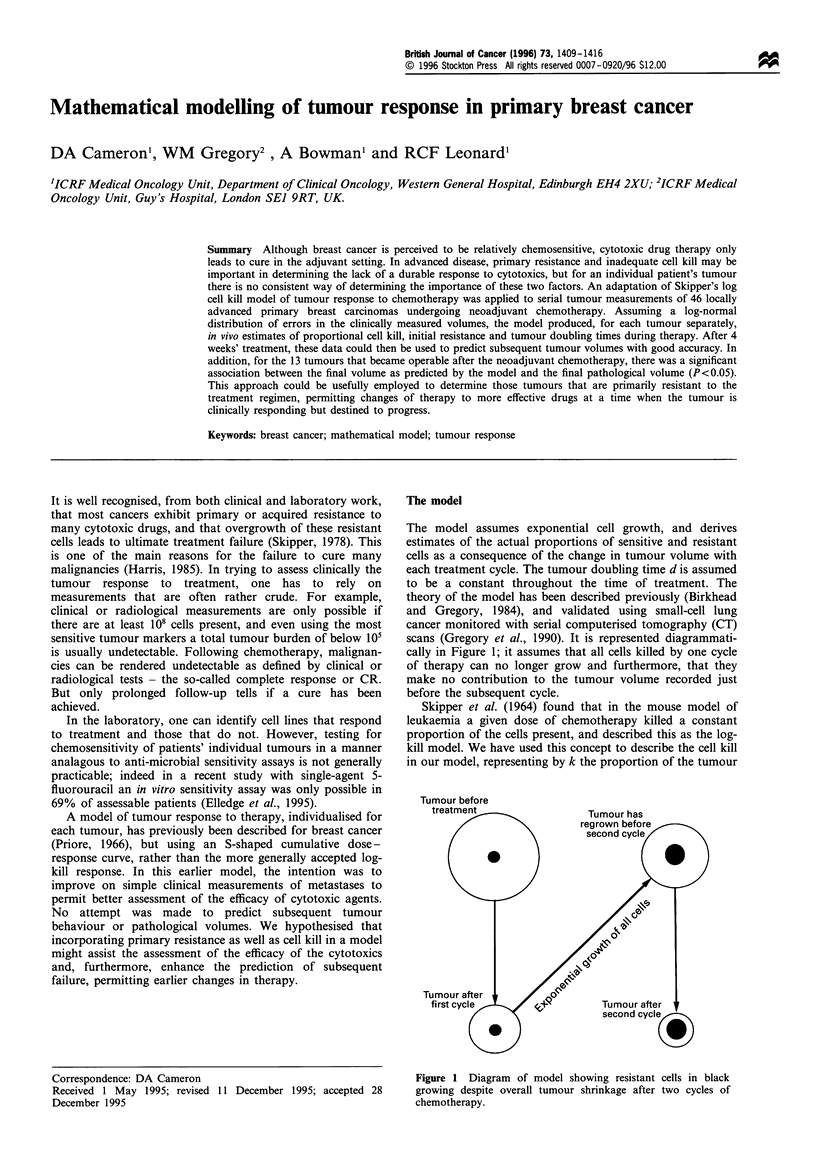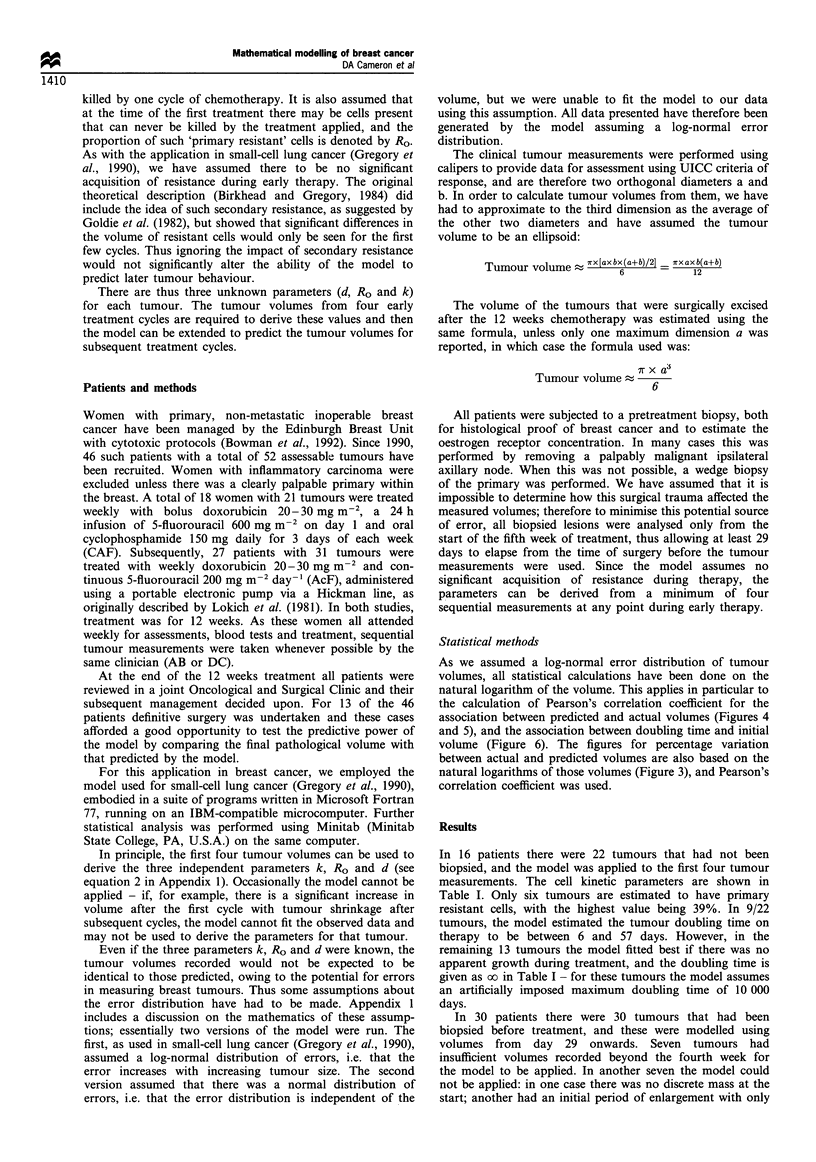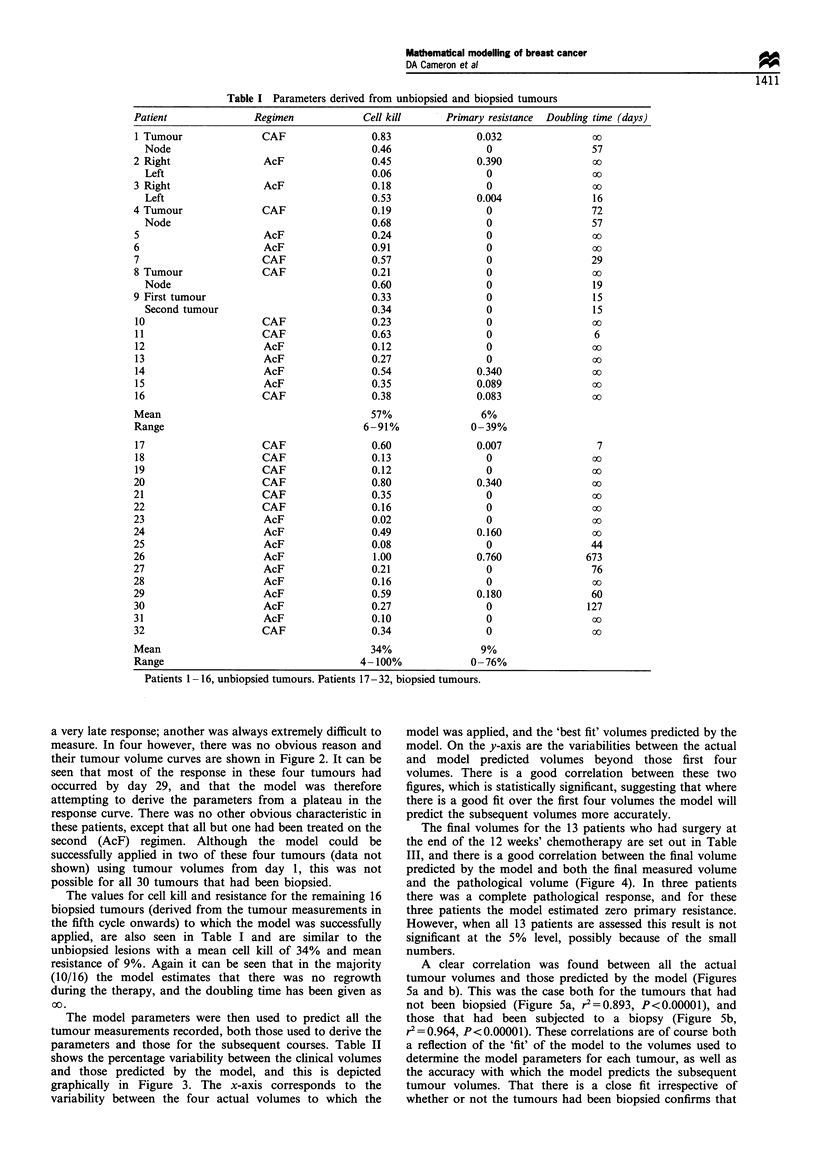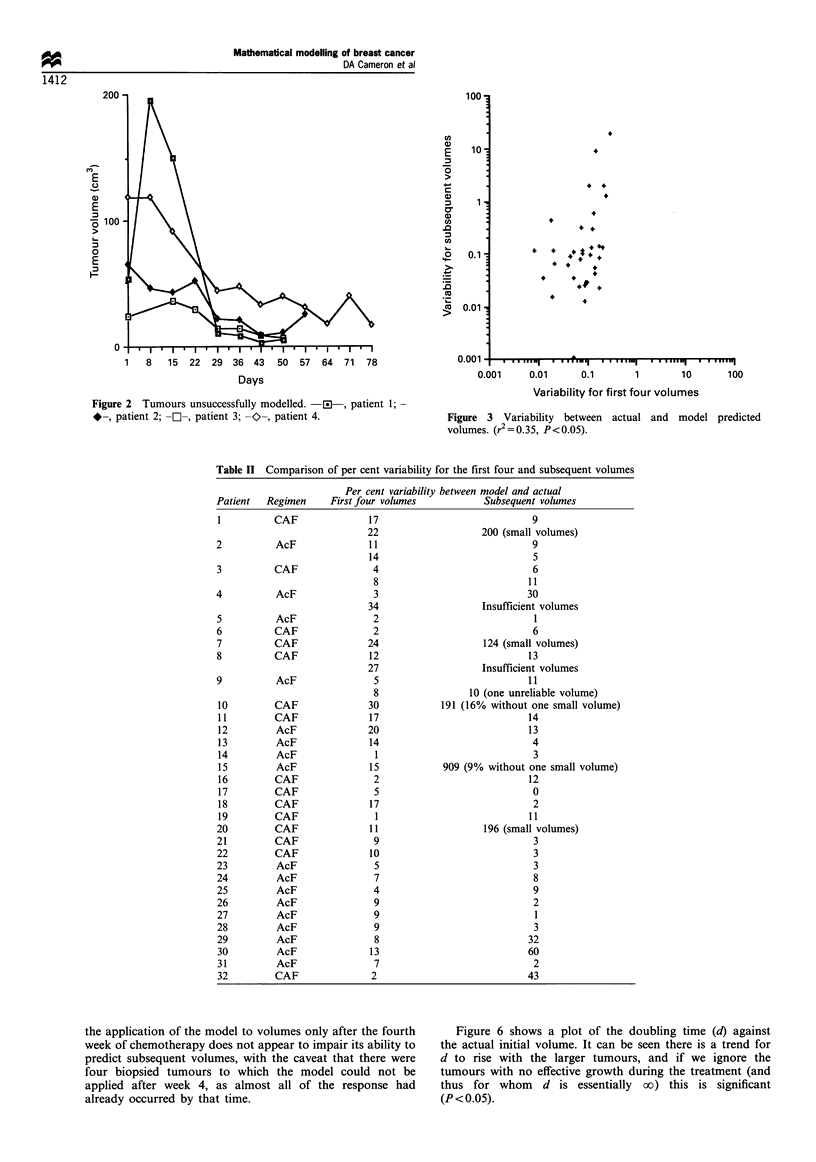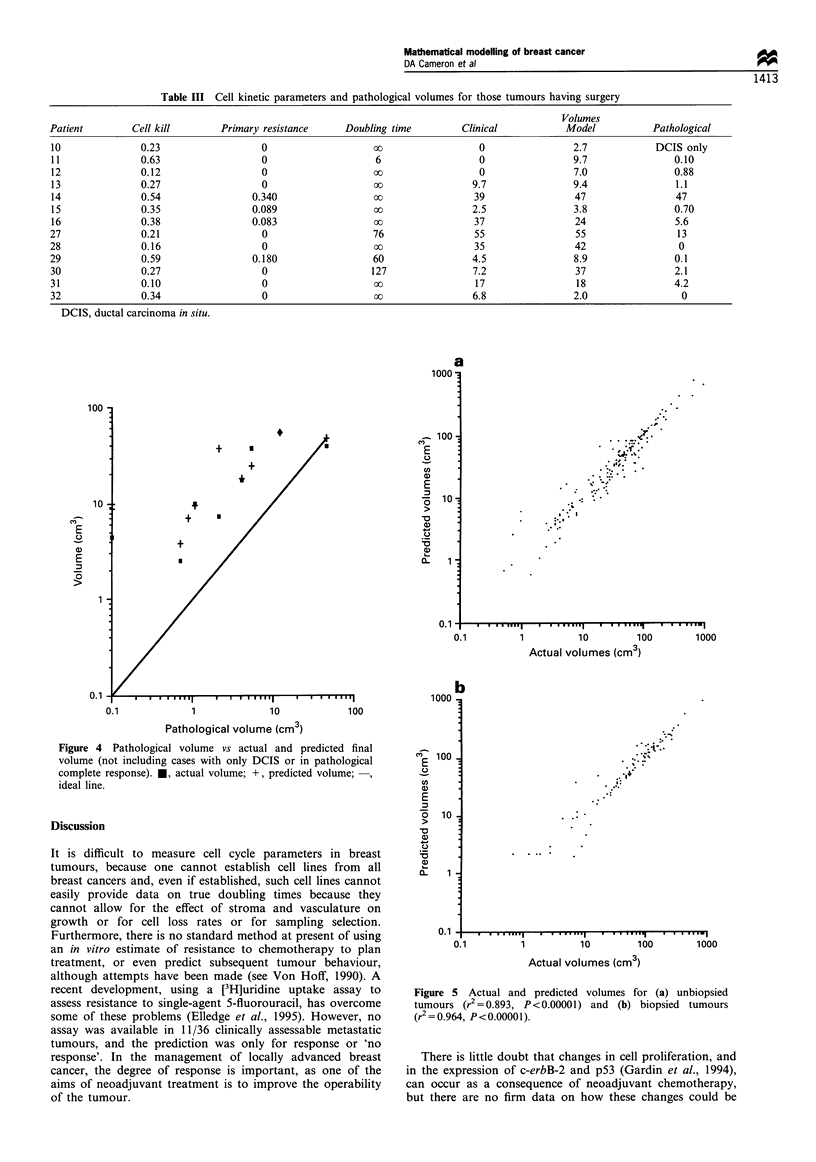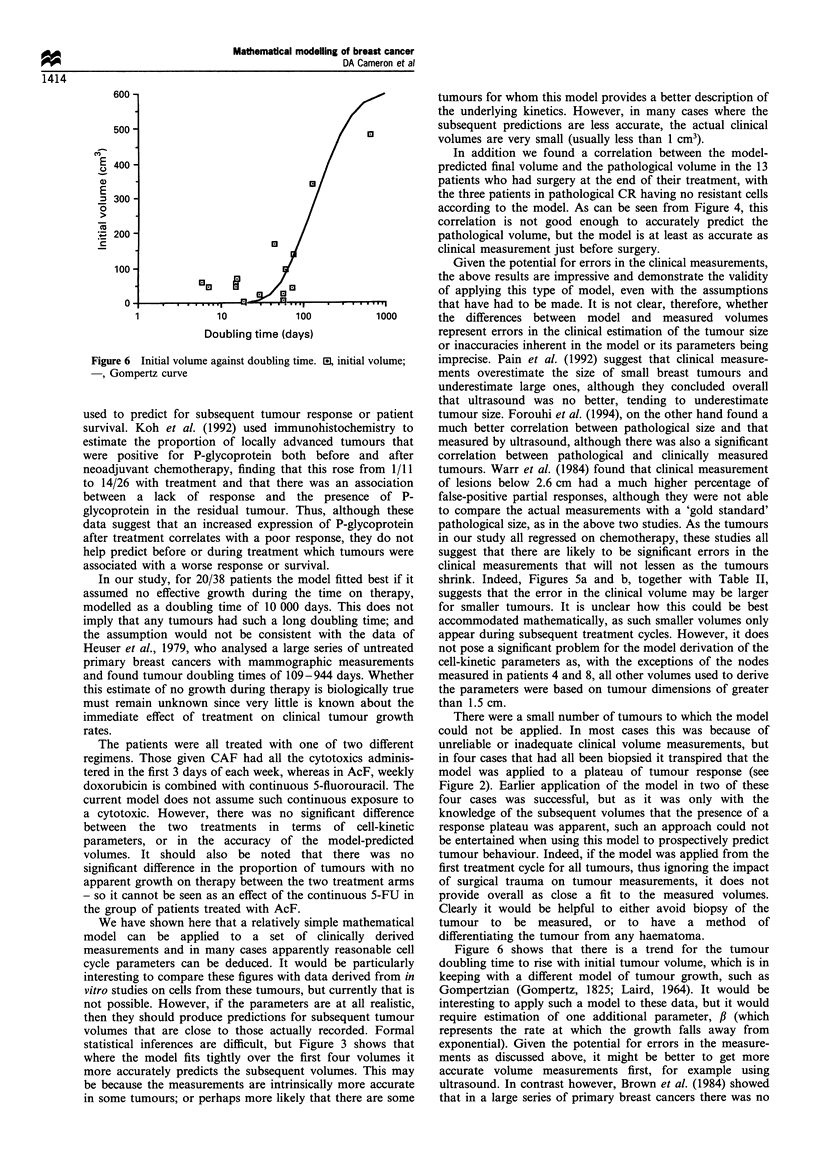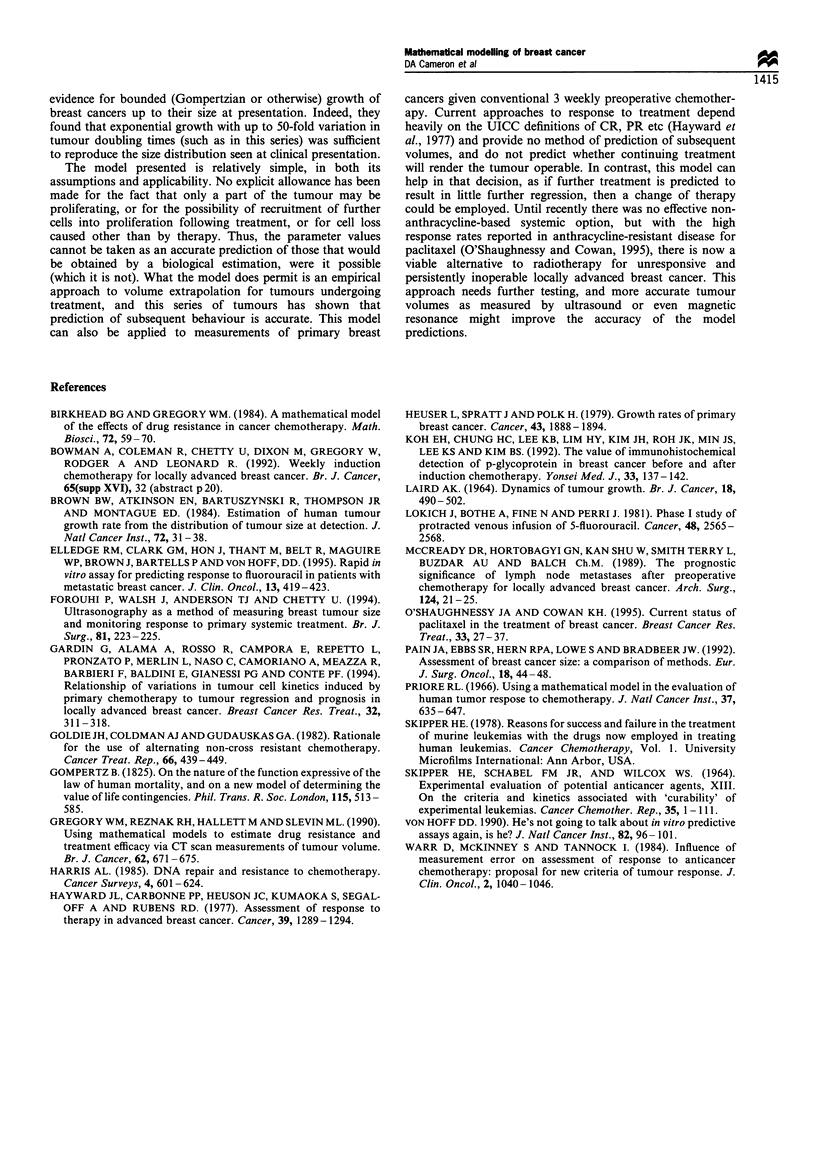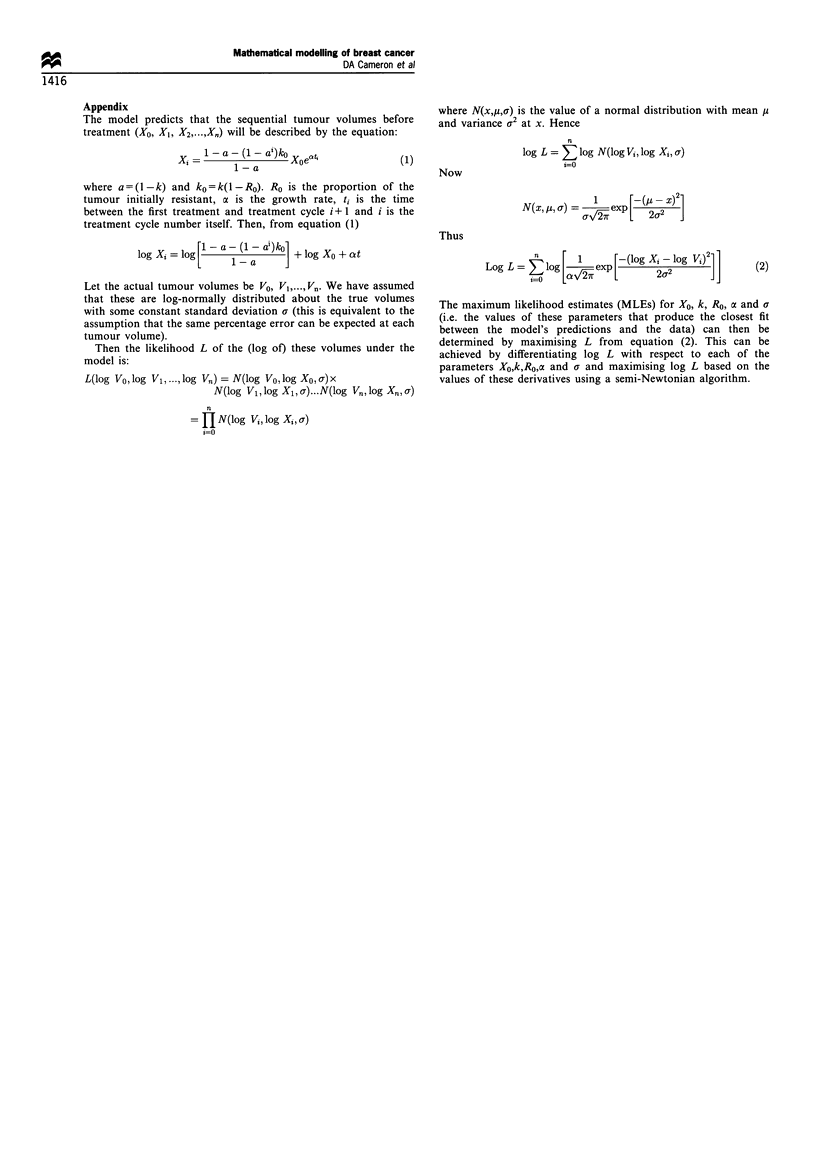# Mathematical modelling of tumour response in primary breast cancer.

**DOI:** 10.1038/bjc.1996.267

**Published:** 1996-06

**Authors:** D. A. Cameron, W. M. Gregory, A. Bowman, R. C. Leonard

**Affiliations:** ICRF Medical Oncology Unit, Department of Clinical Oncology, Western General Hospital, Edinburgh, UK.

## Abstract

Although breast cancer is perceived to be relatively chemosensitive, cytotoxic drug therapy only leads to cure in the adjuvant setting. In advanced disease, primary resistance and inadequate cell kill may be important in determining the lack of a durable response to cytotoxics, but for an individual patient's tumour there is no consistent way of determining the importance of these two factors. An adaptation of Skipper's log cell kill model of tumour response to chemotherapy was applied to serial tumour measurements of 46 locally advanced primary breast carcinomas undergoing neoadjuvant chemotherapy. Assuming a log-normal distribution of errors in the clinically measured volumes, the model produced, for each tumour separately, in vivo estimates of proportional cell kill, initial resistance and tumour doubling times during therapy. After 4 weeks' treatment, these data could then be used to predict subsequent tumour volumes with good accuracy. In addition, for the 13 tumours that became operable after the neoadjuvant chemotherapy, there was a significant association between the final volume as predicted by the model and the final pathological volume (P < 0.05). This approach could be usefully employed to determine those tumours that are primarily resistant to the treatment regimen, permitting changes of therapy to more effective drugs at a time when the tumour is clinically responding but destined to progress.


British Journal of Cancer (1996) 73, 1409-1416
© 1996 Stockton Press All rights reserved 0007-0920/96 $12.00

### Mathematical modelling of tumour response in primary breast cancer

DA Cameron[1], WM Gregory[2], A Bowman[1] and RCF Leonard[1]

[1]*ICRF Medical Oncology Unit, Department of Clinical Oncology, Western General Hospital, Edinburgh EH4 2XU;* [2]*ICRF Medical Oncology Unit, Guy's Hospital, London SE1 9RT, UK.*

**Summary** Although breast cancer is perceived to be relatively chemosensitive, cytotoxic drug therapy only leads to cure in the adjuvant setting. In advanced disease, primary resistance and inadequate cell kill may be important in determining the lack of a durable response to cytotoxics, but for an individual patient's tumour there is no consistent way of determining the importance of these two factors. An adaptation of Skipper's log cell kill model of tumour response to chemotherapy was applied to serial tumour measurements of 46 locally advanced primary breast carcinomas undergoing neoadjuvant chemotherapy. Assuming a log-normal distribution of errors in the clinically measured volumes, the model produced, for each tumour separately, *in vivo* estimates of proportional cell kill, initial resistance and tumour doubling times during therapy. After 4 weeks' treatment, these data could then be used to predict subsequent tumour volumes with good accuracy. In addition, for the 13 tumours that became operable after the neoadjuvant chemotherapy, there was a significant association between the final volume as predicted by the model and the final pathological volume ($P<0.05$). This approach could be usefully employed to determine those tumours that are primarily resistant to the treatment regimen, permitting changes of therapy to more effective drugs at a time when the tumour is clinically responding but destined to progress.

**Keywords:** breast cancer; mathematical model; tumour response

It is well recognised, from both clinical and laboratory work, that most cancers exhibit primary or acquired resistance to many cytotoxic drugs, and that overgrowth of these resistant cells leads to ultimate treatment failure (Skipper, 1978). This is one of the main reasons for the failure to cure many malignancies (Harris, 1985). In trying to assess clinically the tumour response to treatment, one has to rely on measurements that are often rather crude. For example, clinical or radiological measurements are only possible if there are at least $10^8$ cells present, and even using the most sensitive tumour markers a total tumour burden of below $10^5$ is usually undetectable. Following chemotherapy, malignancies can be rendered undetectable as defined by clinical or radiological tests – the so-called complete response or CR. But only prolonged follow-up tells if a cure has been achieved.

In the laboratory, one can identify cell lines that respond to treatment and those that do not. However, testing for chemosensitivity of patients' individual tumours in a manner analagous to anti-microbial sensitivity assays is not generally practicable; indeed in a recent study with single-agent 5-fluorouracil an *in vitro* sensitivity assay was only possible in 69% of assessable patients (Elledge *et al.*, 1995).

A model of tumour response to therapy, individualised for each tumour, has previously been described for breast cancer (Priore, 1966), but using an S-shaped cumulative dose–response curve, rather than the more generally accepted log-kill response. In this earlier model, the intention was to improve on simple clinical measurements of metastases to permit better assessment of the efficacy of cytotoxic agents. No attempt was made to predict subsequent tumour behaviour or pathological volumes. We hypothesised that incorporating primary resistance as well as cell kill in a model might assist the assessment of the efficacy of the cytotoxics and, furthermore, enhance the prediction of subsequent failure, permitting earlier changes in therapy.

### The model

The model assumes exponential cell growth, and derives estimates of the actual proportions of sensitive and resistant cells as a consequence of the change in tumour volume with each treatment cycle. The tumour doubling time $d$ is assumed to be a constant throughout the time of treatment. The theory of the model has been described previously (Birkhead and Gregory, 1984), and validated using small-cell lung cancer monitored with serial computerised tomography (CT) scans (Gregory *et al.*, 1990). It is represented diagrammatically in Figure 1; it assumes that all cells killed by one cycle of therapy can no longer grow and furthermore, that they make no contribution to the tumour volume recorded just before the subsequent cycle.

Skipper *et al.* (1964) found that in the mouse model of leukaemia a given dose of chemotherapy killed a constant proportion of the cells present, and described this as the log-kill model. We have used this concept to describe the cell kill in our model, representing by $k$ the proportion of the tumour

**Figure 1** Diagram of model showing resistant cells in black growing despite overall tumour shrinkage after two cycles of chemotherapy.

Correspondence: DA Cameron
Received 1 May 1995; revised 11 December 1995; accepted 28 December 1995

killed by one cycle of chemotherapy. It is also assumed that at the time of the first treatment there may be cells present that can never be killed by the treatment applied, and the proportion of such 'primary resistant' cells is denoted by $R_O$. As with the application in small-cell lung cancer (Gregory et al., 1990), we have assumed there to be no significant acquisition of resistance during early therapy. The original theoretical description (Birkhead and Gregory, 1984) did include the idea of such secondary resistance, as suggested by Goldie et al. (1982), but showed that significant differences in the volume of resistant cells would only be seen for the first few cycles. Thus ignoring the impact of secondary resistance would not significantly alter the ability of the model to predict later tumour behaviour.

There are thus three unknown parameters ($d$, $R_O$ and $k$) for each tumour. The tumour volumes from four early treatment cycles are required to derive these values and then the model can be extended to predict the tumour volumes for subsequent treatment cycles.

### Patients and methods

Women with primary, non-metastatic inoperable breast cancer have been managed by the Edinburgh Breast Unit with cytotoxic protocols (Bowman et al., 1992). Since 1990, 46 such patients with a total of 52 assessable tumours have been recruited. Women with inflammatory carcinoma were excluded unless there was a clearly palpable primary within the breast. A total of 18 women with 21 tumours were treated weekly with bolus doxorubicin 20–30 mg $m^{-2}$, a 24 h infusion of 5-fluorouracil 600 mg $m^{-2}$ on day 1 and oral cyclophosphamide 150 mg daily for 3 days of each week (CAF). Subsequently, 27 patients with 31 tumours were treated with weekly doxorubicin 20–30 mg $m^{-2}$ and continuous 5-fluorouracil 200 mg $m^{-2}$ $day^{-1}$ (AcF), administered using a portable electronic pump via a Hickman line, as originally described by Lokich et al. (1981). In both studies, treatment was for 12 weeks. As these women all attended weekly for assessments, blood tests and treatment, sequential tumour measurements were taken whenever possible by the same clinician (AB or DC).

At the end of the 12 weeks treatment all patients were reviewed in a joint Oncological and Surgical Clinic and their subsequent management decided upon. For 13 of the 46 patients definitive surgery was undertaken and these cases afforded a good opportunity to test the predictive power of the model by comparing the final pathological volume with that predicted by the model.

For this application in breast cancer, we employed the model used for small-cell lung cancer (Gregory et al., 1990), embodied in a suite of programs written in Microsoft Fortran 77, running on an IBM-compatible microcomputer. Further statistical analysis was performed using Minitab (Minitab State College, PA, U.S.A.) on the same computer.

In principle, the first four tumour volumes can be used to derive the three independent parameters $k$, $R_O$ and $d$ (see equation 2 in Appendix 1). Occasionally the model cannot be applied – if, for example, there is a significant increase in volume after the first cycle with tumour shrinkage after subsequent cycles, the model cannot fit the observed data and may not be used to derive the parameters for that tumour.

Even if the three parameters $k$, $R_O$ and $d$ were known, the tumour volumes recorded would not be expected to be identical to those predicted, owing to the potential for errors in measuring breast tumours. Thus some assumptions about the error distribution have had to be made. Appendix 1 includes a discussion on the mathematics of these assumptions; essentially two versions of the model were run. The first, as used in small-cell lung cancer (Gregory et al., 1990), assumed a log-normal distribution of errors, i.e. that the error increases with increasing tumour size. The second version assumed that there was a normal distribution of errors, i.e. that the error distribution is independent of the volume, but we were unable to fit the model to our data using this assumption. All data presented have therefore been generated by the model assuming a log-normal error distribution.

The clinical tumour measurements were performed using calipers to provide data for assessment using UICC criteria of response, and are therefore two orthogonal diameters a and b. In order to calculate tumour volumes from them, we have had to approximate to the third dimension as the average of the other two diameters and have assumed the tumour volume to be an ellipsoid:

$$\text{Tumour volume} \approx \frac{\pi\times[a\times b\times(a+b)/2]}{6} = \frac{\pi\times a\times b(a+b)}{12}$$

The volume of the tumours that were surgically excised after the 12 weeks chemotherapy was estimated using the same formula, unless only one maximum dimension $a$ was reported, in which case the formula used was:

$$\text{Tumour volume} \approx \frac{\pi \times a^3}{6}$$

All patients were subjected to a pretreatment biopsy, both for histological proof of breast cancer and to estimate the oestrogen receptor concentration. In many cases this was performed by removing a palpably malignant ipsilateral axillary node. When this was not possible, a wedge biopsy of the primary was performed. We have assumed that it is impossible to determine how this surgical trauma affected the measured volumes; therefore to minimise this potential source of error, all biopsied lesions were analysed only from the start of the fifth week of treatment, thus allowing at least 29 days to elapse from the time of surgery before the tumour measurements were used. Since the model assumes no significant acquisition of resistance during therapy, the parameters can be derived from a minimum of four sequential measurements at any point during early therapy.

### Statistical methods

As we assumed a log-normal error distribution of tumour volumes, all statistical calculations have been done on the natural logarithm of the volume. This applies in particular to the calculation of Pearson's correlation coefficient for the association between predicted and actual volumes (Figures 4 and 5), and the association between doubling time and initial volume (Figure 6). The figures for percentage variation between actual and predicted volumes are also based on the natural logarithms of those volumes (Figure 3), and Pearson's correlation coefficient was used.

### Results

In 16 patients there were 22 tumours that had not been biopsied, and the model was applied to the first four tumour measurements. The cell kinetic parameters are shown in Table I. Only six tumours are estimated to have primary resistant cells, with the highest value being 39%. In 9/22 tumours, the model estimated the tumour doubling time on therapy to be between 6 and 57 days. However, in the remaining 13 tumours the model fitted best if there was no apparent growth during treatment, and the doubling time is given as $\infty$ in Table I – for these tumours the model assumes an artificially imposed maximum doubling time of 10 000 days.

In 30 patients there were 30 tumours that had been biopsied before treatment, and these were modelled using volumes from day 29 onwards. Seven tumours had insufficient volumes recorded beyond the fourth week for the model to be applied. In another seven the model could not be applied: in one case there was no discrete mass at the start; another had an initial period of enlargement with only

Table I Parameters derived from unbiopsied and biopsied tumours

| Patient | Regimen | Cell kill | Primary resistance | Doubling time (days) |
|---|---|---|---|---|
| 1 Tumour | CAF | 0.83 | 0.032 | ∞ |
| Node | | 0.46 | 0 | 57 |
| 2 Right | AcF | 0.45 | 0.390 | ∞ |
| Left | | 0.06 | 0 | ∞ |
| 3 Right | AcF | 0.18 | 0 | ∞ |
| Left | | 0.53 | 0.004 | 16 |
| 4 Tumour | CAF | 0.19 | 0 | 72 |
| Node | | 0.68 | 0 | 57 |
| 5 | AcF | 0.24 | 0 | ∞ |
| 6 | AcF | 0.91 | 0 | ∞ |
| 7 | CAF | 0.57 | 0 | 29 |
| 8 Tumour | CAF | 0.21 | 0 | ∞ |
| Node | | 0.60 | 0 | 19 |
| 9 First tumour | | 0.33 | 0 | 15 |
| Second tumour | | 0.34 | 0 | 15 |
| 10 | CAF | 0.23 | 0 | ∞ |
| 11 | CAF | 0.63 | 0 | 6 |
| 12 | AcF | 0.12 | 0 | ∞ |
| 13 | AcF | 0.27 | 0 | ∞ |
| 14 | AcF | 0.54 | 0.340 | ∞ |
| 15 | AcF | 0.35 | 0.089 | ∞ |
| 16 | CAF | 0.38 | 0.083 | ∞ |
| Mean | | 57% | 6% | |
| Range | | 6–91% | 0–39% | |
| 17 | CAF | 0.60 | 0.007 | 7 |
| 18 | CAF | 0.13 | 0 | ∞ |
| 19 | CAF | 0.12 | 0 | ∞ |
| 20 | CAF | 0.80 | 0.340 | ∞ |
| 21 | CAF | 0.35 | 0 | ∞ |
| 22 | CAF | 0.16 | 0 | ∞ |
| 23 | AcF | 0.02 | 0 | ∞ |
| 24 | AcF | 0.49 | 0.160 | ∞ |
| 25 | AcF | 0.08 | 0 | 44 |
| 26 | AcF | 1.00 | 0.760 | 673 |
| 27 | AcF | 0.21 | 0 | 76 |
| 28 | AcF | 0.16 | 0 | ∞ |
| 29 | AcF | 0.59 | 0.180 | 60 |
| 30 | AcF | 0.27 | 0 | 127 |
| 31 | AcF | 0.10 | 0 | ∞ |
| 32 | CAF | 0.34 | 0 | ∞ |
| Mean | | 34% | 9% | |
| Range | | 4–100% | 0–76% | |

Patients 1–16, unbiopsied tumours. Patients 17–32, biopsied tumours.

a very late response; another was always extremely difficult to measure. In four however, there was no obvious reason and their tumour volume curves are shown in Figure 2. It can be seen that most of the response in these four tumours had occurred by day 29, and that the model was therefore attempting to derive the parameters from a plateau in the response curve. There was no other obvious characteristic in these patients, except that all but one had been treated on the second (AcF) regimen. Although the model could be successfully applied in two of these four tumours (data not shown) using tumour volumes from day 1, this was not possible for all 30 tumours that had been biopsied.

The values for cell kill and resistance for the remaining 16 biopsied tumours (derived from the tumour measurements in the fifth cycle onwards) to which the model was successfully applied, are also seen in Table I and are similar to the unbiopsied lesions with a mean cell kill of 34% and mean resistance of 9%. Again it can be seen that in the majority (10/16) the model estimates that there was no regrowth during the therapy, and the doubling time has been given as ∞.

The model parameters were then used to predict all the tumour measurements recorded, both those used to derive the parameters and those for the subsequent courses. Table II shows the percentage variability between the clinical volumes and those predicted by the model, and this is depicted graphically in Figure 3. The $x$-axis corresponds to the variability between the four actual volumes to which the model was applied, and the 'best fit' volumes predicted by the model. On the $y$-axis are the variabilities between the actual and model predicted volumes beyond those first four volumes. There is a good correlation between these two figures, which is statistically significant, suggesting that where there is a good fit over the first four volumes the model will predict the subsequent volumes more accurately.

The final volumes for the 13 patients who had surgery at the end of the 12 weeks' chemotherapy are set out in Table III, and there is a good correlation between the final volume predicted by the model and both the final measured volume and the pathological volume (Figure 4). In three patients there was a complete pathological response, and for these three patients the model estimated zero primary resistance. However, when all 13 patients are assessed this result is not significant at the 5% level, possibly because of the small numbers.

A clear correlation was found between all the actual tumour volumes and those predicted by the model (Figures 5a and b). This was the case both for the tumours that had not been biopsied (Figure 5a, $r^2=0.893$, $P<0.00001$), and those that had been subjected to a biopsy (Figure 5b, $r^2=0.964$, $P<0.00001$). These correlations are of course both a reflection of the 'fit' of the model to the volumes used to determine the model parameters for each tumour, as well as the accuracy with which the model predicts the subsequent tumour volumes. That there is a close fit irrespective of whether or not the tumours had been biopsied confirms that

**Figure 2** Tumours unsuccessfully modelled. —▣—, patient 1; –◆–, patient 2; –□–, patient 3; –◇–, patient 4.

**Figure 3** Variability between actual and model predicted volumes. ($r^2=0.35$, $P<0.05$).

**Table II** Comparison of per cent variability for the first four and subsequent volumes

| Patient | Regimen | *Per cent variability between model and actual* First four volumes | Subsequent volumes |
|---|---|---|---|
| 1 | CAF | 17 | 9 |
| | | 22 | 200 (small volumes) |
| 2 | AcF | 11 | 9 |
| | | 14 | 5 |
| 3 | CAF | 4 | 6 |
| | | 8 | 11 |
| 4 | AcF | 3 | 30 |
| | | 34 | Insufficient volumes |
| 5 | AcF | 2 | 1 |
| 6 | CAF | 2 | 6 |
| 7 | CAF | 24 | 124 (small volumes) |
| 8 | CAF | 12 | 13 |
| | | 27 | Insufficient volumes |
| 9 | AcF | 5 | 11 |
| | | 8 | 10 (one unreliable volume) |
| 10 | CAF | 30 | 191 (16% without one small volume) |
| 11 | CAF | 17 | 14 |
| 12 | AcF | 20 | 13 |
| 13 | AcF | 14 | 4 |
| 14 | AcF | 1 | 3 |
| 15 | AcF | 15 | 909 (9% without one small volume) |
| 16 | CAF | 2 | 12 |
| 17 | CAF | 5 | 0 |
| 18 | CAF | 17 | 2 |
| 19 | CAF | 1 | 11 |
| 20 | CAF | 11 | 196 (small volumes) |
| 21 | CAF | 9 | 3 |
| 22 | CAF | 10 | 3 |
| 23 | AcF | 5 | 3 |
| 24 | AcF | 7 | 8 |
| 25 | AcF | 4 | 9 |
| 26 | AcF | 9 | 2 |
| 27 | AcF | 9 | 1 |
| 28 | AcF | 9 | 3 |
| 29 | AcF | 8 | 32 |
| 30 | AcF | 13 | 60 |
| 31 | AcF | 7 | 2 |
| 32 | CAF | 2 | 43 |

the application of the model to volumes only after the fourth week of chemotherapy does not appear to impair its ability to predict subsequent volumes, with the caveat that there were four biopsied tumours to which the model could not be applied after week 4, as almost all of the response had already occurred by that time.

Figure 6 shows a plot of the doubling time ($d$) against the actual initial volume. It can be seen there is a trend for $d$ to rise with the larger tumours, and if we ignore the tumours with no effective growth during the treatment (and thus for whom $d$ is essentially $\infty$) this is significant ($P<0.05$).

Table III Cell kinetic parameters and pathological volumes for those tumours having surgery

| Patient | Cell kill | Primary resistance | Doubling time | Volumes Clinical | Volumes Model | Volumes Pathological |
|---|---|---|---|---|---|---|
| 10 | 0.23 | 0 | ∞ | 0 | 2.7 | DCIS only |
| 11 | 0.63 | 0 | 6 | 0 | 9.7 | 0.10 |
| 12 | 0.12 | 0 | ∞ | 0 | 7.0 | 0.88 |
| 13 | 0.27 | 0 | ∞ | 9.7 | 9.4 | 1.1 |
| 14 | 0.54 | 0.340 | ∞ | 39 | 47 | 47 |
| 15 | 0.35 | 0.089 | ∞ | 2.5 | 3.8 | 0.70 |
| 16 | 0.38 | 0.083 | ∞ | 37 | 24 | 5.6 |
| 27 | 0.21 | 0 | 76 | 55 | 55 | 13 |
| 28 | 0.16 | 0 | ∞ | 35 | 42 | 0 |
| 29 | 0.59 | 0.180 | 60 | 4.5 | 8.9 | 0.1 |
| 30 | 0.27 | 0 | 127 | 7.2 | 37 | 2.1 |
| 31 | 0.10 | 0 | ∞ | 17 | 18 | 4.2 |
| 32 | 0.34 | 0 | ∞ | 6.8 | 2.0 | 0 |

DCIS, ductal carcinoma *in situ*.

**Figure 4** Pathological volume *vs* actual and predicted final volume (not including cases with only DCIS or in pathological complete response). ■, actual volume; +, predicted volume; —, ideal line.

### Discussion

It is difficult to measure cell cycle parameters in breast tumours, because one cannot establish cell lines from all breast cancers and, even if established, such cell lines cannot easily provide data on true doubling times because they cannot allow for the effect of stroma and vasculature on growth or for cell loss rates or for sampling selection. Furthermore, there is no standard method at present of using an *in vitro* estimate of resistance to chemotherapy to plan treatment, or even predict subsequent tumour behaviour, although attempts have been made (see Von Hoff, 1990). A recent development, using a [$^3$H]uridine uptake assay to assess resistance to single-agent 5-fluorouracil, has overcome some of these problems (Elledge *et al.*, 1995). However, no assay was available in 11/36 clinically assessable metastatic tumours, and the prediction was only for response or 'no response'. In the management of locally advanced breast cancer, the degree of response is important, as one of the aims of neoadjuvant treatment is to improve the operability of the tumour.

**Figure 5** Actual and predicted volumes for (**a**) unbiopsied tumours ($r^2$=0.893, $P$<0.00001) and (**b**) biopsied tumours ($r^2$=0.964, $P$<0.00001).

There is little doubt that changes in cell proliferation, and in the expression of c-*erb*B-2 and p53 (Gardin *et al.*, 1994), can occur as a consequence of neoadjuvant chemotherapy, but there are no firm data on how these changes could be

**Figure 6** Initial volume against doubling time. ▣, initial volume; —, Gompertz curve used to predict for subsequent tumour response or patient survival. Koh *et al.* (1992) used immunohistochemistry to estimate the proportion of locally advanced tumours that were positive for P-glycoprotein both before and after neoadjuvant chemotherapy, finding that this rose from 1/11 to 14/26 with treatment and that there was an association between a lack of response and the presence of P-glycoprotein in the residual tumour. Thus, although these data suggest that an increased expression of P-glycoprotein after treatment correlates with a poor response, they do not help predict before or during treatment which tumours were associated with a worse response or survival.

In our study, for 20/38 patients the model fitted best if it assumed no effective growth during the time on therapy, modelled as a doubling time of 10 000 days. This does not imply that any tumours had such a long doubling time; and the assumption would not be consistent with the data of Heuser *et al.*, 1979, who analysed a large series of untreated primary breast cancers with mammographic measurements and found tumour doubling times of 109–944 days. Whether this estimate of no growth during therapy is biologically true must remain unknown since very little is known about the immediate effect of treatment on clinical tumour growth rates.

The patients were all treated with one of two different regimens. Those given CAF had all the cytotoxics administered in the first 3 days of each week, whereas in AcF, weekly doxorubicin is combined with continuous 5-fluorouracil. The current model does not assume such continuous exposure to a cytotoxic. However, there was no significant difference between the two treatments in terms of cell-kinetic parameters, or in the accuracy of the model-predicted volumes. It should also be noted that there was no significant difference in the proportion of tumours with no apparent growth on therapy between the two treatment arms – so it cannot be seen as an effect of the continuous 5-FU in the group of patients treated with AcF.

We have shown here that a relatively simple mathematical model can be applied to a set of clinically derived measurements and in many cases apparently reasonable cell cycle parameters can be deduced. It would be particularly interesting to compare these figures with data derived from *in vitro* studies on cells from these tumours, but currently that is not possible. However, if the parameters are at all realistic, then they should produce predictions for subsequent tumour volumes that are close to those actually recorded. Formal statistical inferences are difficult, but Figure 3 shows that where the model fits tightly over the first four volumes it more accurately predicts the subsequent volumes. This may be because the measurements are intrinsically more accurate in some tumours; or perhaps more likely that there are some tumours for whom this model provides a better description of the underlying kinetics. However, in many cases where the subsequent predictions are less accurate, the actual clinical volumes are very small (usually less than 1 $cm^3$).

In addition we found a correlation between the model-predicted final volume and the pathological volume in the 13 patients who had surgery at the end of their treatment, with the three patients in pathological CR having no resistant cells according to the model. As can be seen from Figure 4, this correlation is not good enough to accurately predict the pathological volume, but the model is at least as accurate as clinical measurement just before surgery.

Given the potential for errors in the clinical measurements, the above results are impressive and demonstrate the validity of applying this type of model, even with the assumptions that have had to be made. It is not clear, therefore, whether the differences between model and measured volumes represent errors in the clinical estimation of the tumour size or inaccuracies inherent in the model or its parameters being imprecise. Pain *et al.* (1992) suggest that clinical measurements overestimate the size of small breast tumours and underestimate large ones, although they concluded overall that ultrasound was no better, tending to underestimate tumour size. Forouhi *et al.* (1994), on the other hand found a much better correlation between pathological size and that measured by ultrasound, although there was also a significant correlation between pathological and clinically measured tumours. Warr *et al.* (1984) found that clinical measurement of lesions below 2.6 cm had a much higher percentage of false-positive partial responses, although they were not able to compare the actual measurements with a 'gold standard' pathological size, as in the above two studies. As the tumours in our study all regressed on chemotherapy, these studies all suggest that there are likely to be significant errors in the clinical measurements that will not lessen as the tumours shrink. Indeed, Figures 5a and b, together with Table II, suggests that the error in the clinical volume may be larger for smaller tumours. It is unclear how this could be best accommodated mathematically, as such smaller volumes only appear during subsequent treatment cycles. However, it does not pose a significant problem for the model derivation of the cell-kinetic parameters as, with the exceptions of the nodes measured in patients 4 and 8, all other volumes used to derive the parameters were based on tumour dimensions of greater than 1.5 cm.

There were a small number of tumours to which the model could not be applied. In most cases this was because of unreliable or inadequate clinical volume measurements, but in four cases that had all been biopsied it transpired that the model was applied to a plateau of tumour response (see Figure 2). Earlier application of the model in two of these four cases was successful, but as it was only with the knowledge of the subsequent volumes that the presence of a response plateau was apparent, such an approach could not be entertained when using this model to prospectively predict tumour behaviour. Indeed, if the model was applied from the first treatment cycle for all tumours, thus ignoring the impact of surgical trauma on tumour measurements, it does not provide overall as close a fit to the measured volumes. Clearly it would be helpful to either avoid biopsy of the tumour to be measured, or to have a method of differentiating the tumour from any haematoma.

Figure 6 shows that there is a trend for the tumour doubling time to rise with initial tumour volume, which is in keeping with a different model of tumour growth, such as Gompertzian (Gompertz, 1825; Laird, 1964). It would be interesting to apply such a model to these data, but it would require estimation of one additional parameter, $\beta$ (which represents the rate at which the growth falls away from exponential). Given the potential for errors in the measurements as discussed above, it might be better to get more accurate volume measurements first, for example using ultrasound. In contrast however, Brown *et al.* (1984) showed that in a large series of primary breast cancers there was no evidence for bounded (Gompertzian or otherwise) growth of breast cancers up to their size at presentation. Indeed, they found that exponential growth with up to 50-fold variation in tumour doubling times (such as in this series) was sufficient to reproduce the size distribution seen at clinical presentation.

The model presented is relatively simple, in both its assumptions and applicability. No explicit allowance has been made for the fact that only a part of the tumour may be proliferating, or for the possibility of recruitment of further cells into proliferation following treatment, or for cell loss caused other than by therapy. Thus, the parameter values cannot be taken as an accurate prediction of those that would be obtained by a biological estimation, were it possible (which it is not). What the model does permit is an empirical approach to volume extrapolation for tumours undergoing treatment, and this series of tumours has shown that prediction of subsequent behaviour is accurate. This model can also be applied to measurements of primary breast cancers given conventional 3 weekly preoperative chemotherapy. Current approaches to response to treatment depend heavily on the UICC definitions of CR, PR etc (Hayward *et al.*, 1977) and provide no method of prediction of subsequent volumes, and do not predict whether continuing treatment will render the tumour operable. In contrast, this model can help in that decision, as if further treatment is predicted to result in little further regression, then a change of therapy could be employed. Until recently there was no effective non-anthracycline-based systemic option, but with the high response rates reported in anthracycline-resistant disease for paclitaxel (O'Shaughnessy and Cowan, 1995), there is now a viable alternative to radiotherapy for unresponsive and persistently inoperable locally advanced breast cancer. This approach needs further testing, and more accurate tumour volumes as measured by ultrasound or even magnetic resonance might improve the accuracy of the model predictions.

### References

BIRKHEAD BG AND GREGORY WM. (1984). A mathematical model of the effects of drug resistance in cancer chemotherapy. *Math. Biosci.*, **72**, 59–70.

BOWMAN A, COLEMAN R, CHETTY U, DIXON M, GREGORY W, RODGER A AND LEONARD R. (1992). Weekly induction chemotherapy for locally advanced breast cancer. *Br. J. Cancer*, **65(supp XVI)**, 32 (abstract p 20).

BROWN BW, ATKINSON EN, BARTUSZYNSKI R, THOMPSON JR AND MONTAGUE ED. (1984). Estimation of human tumour growth rate from the distribution of tumour size at detection. *J. Natl Cancer Inst.*, **72**, 31–38.

ELLEDGE RM, CLARK GM, HON J, THANT M, BELT R, MAGUIRE WP, BROWN J, BARTELLS P AND VON HOFF, DD. (1995). Rapid *in vitro* assay for predicting response to fluorouracil in patients with metastatic breast cancer. *J. Clin. Oncol.*, **13**, 419–423.

FOROUHI P, WALSH J, ANDERSON TJ AND CHETTY U. (1994). Ultrasonography as a method of measuring breast tumour size and monitoring response to primary systemic treatment. *Br. J. Surg.*, **81**, 223–225.

GARDIN G, ALAMA A, ROSSO R, CAMPORA E, REPETTO L, PRONZATO P, MERLIN L, NASO C, CAMORIANO A, MEAZZA R, BARBIERI F, BALDINI E, GIANESSI PG AND CONTE PF. (1994). Relationship of variations in tumour cell kinetics induced by primary chemotherapy to tumour regression and prognosis in locally advanced breast cancer. *Breast Cancer Res. Treat.*, **32**, 311–318.

GOLDIE JH, COLDMAN AJ AND GUDAUSKAS GA. (1982). Rationale for the use of alternating non-cross resistant chemotherapy. *Cancer Treat. Rep.*, **66**, 439–449.

GOMPERTZ B. (1825). On the nature of the function expressive of the law of human mortality, and on a new model of determining the value of life contingencies. *Phil. Trans. R. Soc. London*, **115**, 513–585.

GREGORY WM, REZNAK RH, HALLETT M AND SLEVIN ML. (1990). Using mathematical models to estimate drug resistance and treatment efficacy via CT scan measurements of tumour volume. *Br. J. Cancer*, **62**, 671–675.

HARRIS AL. (1985). DNA repair and resistance to chemotherapy. *Cancer Surveys*, **4**, 601–624.

HAYWARD JL, CARBONNE PP, HEUSON JC, KUMAOKA S, SEGALOFF A AND RUBENS RD. (1977). Assessment of response to therapy in advanced breast cancer. *Cancer*, **39**, 1289–1294.

HEUSER L, SPRATT J AND POLK H. (1979). Growth rates of primary breast cancer. *Cancer*, **43**, 1888–1894.

KOH EH, CHUNG HC, LEE KB, LIM HY, KIM JH, ROH JK, MIN JS, LEE KS AND KIM BS. (1992). The value of immunohistochemical detection of p-glycoprotein in breast cancer before and after induction chemotherapy. *Yonsei Med. J.*, **33**, 137–142.

LAIRD AK. (1964). Dynamics of tumour growth. *Br. J. Cancer*, **18**, 490–502.

LOKICH J, BOTHE A, FINE N AND PERRI J. 1981). Phase I study of protracted venous infusion of 5-fluorouracil. *Cancer*, **48**, 2565–2568.

MCCREADY DR, HORTOBAGYI GN, KAN SHU W, SMITH TERRY L, BUZDAR AU AND BALCH Ch.M. (1989). The prognostic significance of lymph node metastases after preoperative chemotherapy for locally advanced breast cancer. *Arch. Surg.*, **124**, 21–25.

O'SHAUGHNESSY JA AND COWAN KH. (1995). Current status of paclitaxel in the treatment of breast cancer. *Breast Cancer Res. Treat.*, **33**, 27–37.

PAIN JA, EBBS SR, HERN RPA, LOWE S AND BRADBEER JW. (1992). Assessment of breast cancer size: a comparison of methods. *Eur. J. Surg. Oncol.*, **18**, 44–48.

PRIORE RL. (1966). Using a mathematical model in the evaluation of human tumor respose to chemotherapy. *J. Natl Cancer Inst.*, **37**, 635–647.

SKIPPER HE. (1978). Reasons for success and failure in the treatment of murine leukemias with the drugs now employed in treating human leukemias. *Cancer Chemotherapy*, Vol. 1. University Microfilms International: Ann Arbor, USA.

SKIPPER HE, SCHABEL FM JR, AND WILCOX WS. (1964). Experimental evaluation of potential anticancer agents, XIII. On the criteria and kinetics associated with 'curability' of experimental leukemias. *Cancer Chemother. Rep.*, **35**, 1–111.

VON HOFF DD. 1990). He's not going to talk about *in vitro* predictive assays again, is he? *J. Natl Cancer Inst.*, **82**, 96–101.

WARR D, MCKINNEY S AND TANNOCK I. (1984). Influence of measurement error on assessment of response to anticancer chemotherapy: proposal for new criteria of tumour response. *J. Clin. Oncol.*, **2**, 1040–1046.

**Appendix**

The model predicts that the sequential tumour volumes before treatment ($X_0$, $X_1$, $X_2$,...,$X_n$) will be described by the equation:

$$X_i = \frac{1 - a - (1 - a^i)k_0}{1 - a} X_0 e^{\alpha t_i} \qquad (1)$$

where $a = (1-k)$ and $k_0 = k(1-R_0)$. $R_0$ is the proportion of the tumour initially resistant, $\alpha$ is the growth rate, $t_i$ is the time between the first treatment and treatment cycle $i+1$ and $i$ is the treatment cycle number itself. Then, from equation (1)

$$\log X_i = \log\left[\frac{1 - a - (1 - a^i)k_0}{1 - a}\right] + \log X_0 + \alpha t$$

Let the actual tumour volumes be $V_0$, $V_1$,...,$V_n$. We have assumed that these are log-normally distributed about the true volumes with some constant standard deviation $\sigma$ (this is equivalent to the assumption that the same percentage error can be expected at each tumour volume).

Then the likelihood $L$ of the (log of) these volumes under the model is:

$$L(\log V_0, \log V_1, ..., \log V_n) = N(\log V_0, \log X_0, \sigma) \times N(\log V_1, \log X_1, \sigma) ... N(\log V_n, \log X_n, \sigma)$$

$$= \prod_{i=0}^{n} N(\log V_i, \log X_i, \sigma)$$

where $N(x,\mu,\sigma)$ is the value of a normal distribution with mean $\mu$ and variance $\sigma^2$ at $x$. Hence

$$\log L = \sum_{i=0}^{n} \log N(\log V_i, \log X_i, \sigma)$$

Now

$$N(x, \mu, \sigma) = \frac{1}{\sigma\sqrt{2\pi}} \exp\left[\frac{-(\mu - x)^2}{2\sigma^2}\right]$$

Thus

$$\text{Log } L = \sum_{i=0}^{n} \log\left[\frac{1}{\alpha\sqrt{2\pi}} \exp\left[\frac{-(\log X_i - \log V_i)^2}{2\sigma^2}\right]\right] \qquad (2)$$

The maximum likelihood estimates (MLEs) for $X_0$, $k$, $R_0$, $\alpha$ and $\sigma$ (i.e. the values of these parameters that produce the closest fit between the model's predictions and the data) can then be determined by maximising $L$ from equation (2). This can be achieved by differentiating log $L$ with respect to each of the parameters $X_0$,$k$,$R_0$,$\alpha$ and $\sigma$ and maximising log $L$ based on the values of these derivatives using a semi-Newtonian algorithm.