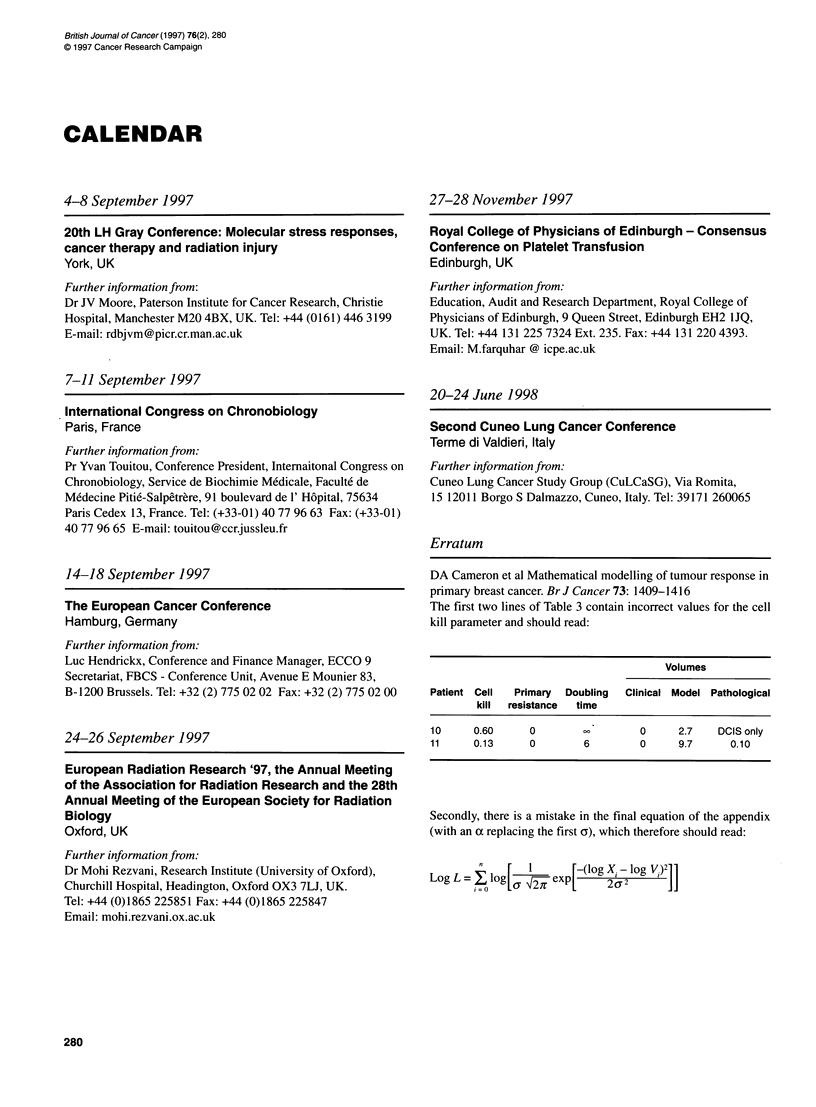# Erratum

**Published:** 1997

**Authors:** 


### *Erratum*

DA Cameron et al Mathematical modelling of tumour response in primary breast cancer. *Br J Cancer* **73**: 1409–1416
The first two lines of Table 3 contain incorrect values for the cell kill parameter and should read:

| Patient | Cell kill | Primary resistance | Doubling time | Volumes Clinical | Model | Pathological |
|---|---|---|---|---|---|---|
| 10 | 0.60 | 0 | ∞* | 0 | 2.7 | DCIS only |
| 11 | 0.13 | 0 | 6 | 0 | 9.7 | 0.10 |

Secondly, there is a mistake in the final equation of the appendix (with an α replacing the first σ), which therefore should read: