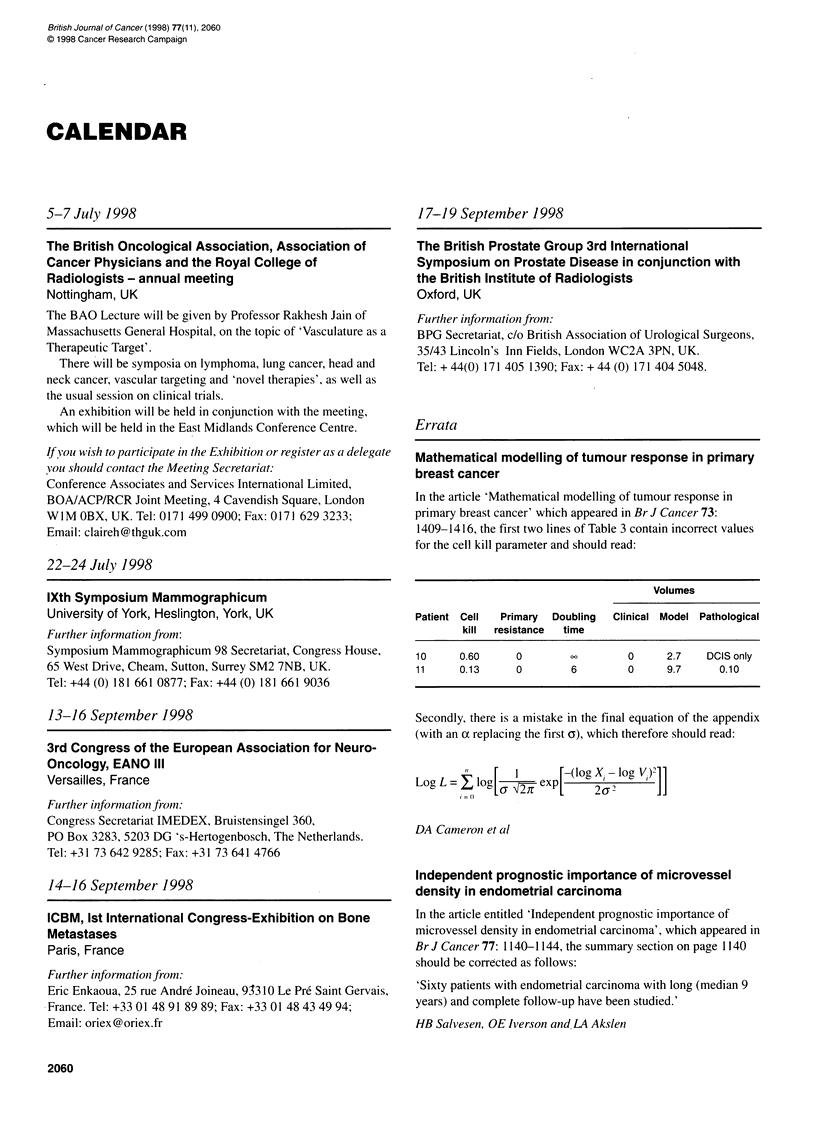# Mathematical modelling of tumour response in primary breast cancer

**Published:** 1998-06

**Authors:** 


### *Errata*

### Mathematical modelling of tumour response in primary breast cancer

In the article 'Mathematical modelling of tumour response in primary breast cancer' which appeared in *Br J Cancer* **73**: 1409–1416, the first two lines of Table 3 contain incorrect values for the cell kill parameter and should read:

| Patient | Cell kill | Primary resistance | Doubling time | Volumes: Clinical | Volumes: Model | Volumes: Pathological |
|---|---|---|---|---|---|---|
| 10 | 0.60 | 0 | ∞ | 0 | 2.7 | DCIS only |
| 11 | 0.13 | 0 | 6 | 0 | 9.7 | 0.10 |

Secondly, there is a mistake in the final equation of the appendix (with an α replacing the first σ), which therefore should read:

$$\text{Log } L = \sum_{i=0}^{n} \log\left[\frac{1}{\sigma\sqrt{2\pi}} \exp\left[\frac{-(\log X_i - \log V_i)^2}{2\sigma^2}\right]\right]$$

*DA Cameron et al*